# Racial And Ethnic Disparities In Buprenorphine Receipt Among Medicare Beneficiaries, 2015–19

**DOI:** 10.1377/hlthaff.2023.00205

**Published:** 2023-10

**Authors:** Jennifer Miles, Peter Treitler, James Lloyd, Hillary Samples, Anais Mahone, Richard Hermida, Sumedha Gupta, Alexandra Duncan, Vanessa Baaklini, Kosali I. Simon, Stephen Crystal

**Affiliations:** Rutgers University, New Brunswick, New Jersey.; Rutgers University.; Rutgers University.; Rutgers University.; Rutgers University.; Rutgers University.; Indiana University–Purdue University Indianapolis, Indianapolis, Indiana.; Pew Charitable Trusts, Washington, D.C.; Pew Charitable Trusts.; Indiana University, Bloomington, Indiana.; Rutgers University.

## Abstract

We examined Medicare Part D claims from the period 2015–19 to identify state and national racial and ethnic disparities in buprenorphine receipt among Medicare disability beneficiaries with diagnosed opioid use disorder or opioid overdose. Racial and ethnic disparities in buprenorphine use remained persistently high during the study period, especially for Black beneficiaries, suggesting the need for targeted interventions and policies.

Highly effective medications for opioid use disorder (OUD), such as buprenorphine, which can be prescribed in office-based settings, are underused among minoritized racial and ethnic populations.^1^ Examining disparities in buprenorphine use within the Medicare population is of particular importance, given high rates of OUD, overdose, and complex comorbidity^2–4^ among Medicare disability beneficiaries ages 18–64 and rising overdose rates among beneficiaries ages 65 and older.^5^ In this study we examined state and national racial and ethnic disparities in buprenorphine receipt among Medicare enrollees with at least one OUD or opioid overdose diagnosis, using data from the period 2015–19. As shown in exhibit 1, during the study period, Black Medicare disability beneficiaries had significantly lower absolute rates of buprenorphine receipt than their White counterparts. National strategies for increasing access to buprenorphine are needed, given increasing opioid-involved overdose mortality among Black adults relative to Whites.^6^ Understanding state variation in buprenorphine disparities can also help identify effective policies and the states in which targeted disparity reduction efforts are most needed.

## Study Data And Methods

We used a 20 percent random sample of Medicare Part D claims in a repeated cross-section design, selecting enrollees with eleven or more months of Medicare fee-for-service coverage in a calendar year. Analyses were limited to buprenorphine because methadone was not covered by Medicare during the study period and low naltrexone use (less than 10 percent) limited statistical power to assess outcomes across racial and ethnic subgroups as defined based on the enhanced Research Triangle Institute (RTI) race and ethnicity variable (Black, Hispanic, Asian/Pacific Islander, American Indian/Alaska Native, and White).^7^ We included people with at least one OUD or opioid overdose diagnosis during the calendar year to capture potential need for treatment, conforming to the Centers for Medicare and Medicaid Services quality measure for “use of pharmacotherapy for opioid use disorder.”^8^ Oral and injectable buprenorphine formulations were identified using National Drug Codes, excluding formulations approved for pain treatment (see online appendix exhibits A1 and A2 for diagnosis codes and search terms).^9^

Annual state-level rates of buprenorphine receipt were calculated as the number of beneficiaries with filled buprenorphine prescriptions divided by the total number of beneficiaries with an OUD or opioid overdose diagnosis in each state, overall, by eligibility category (disability and older adult), and by race and ethnicity. State-level disparity ratios were calculated as the ratio of the proportion of people receiving buprenorphine in each group of racial and ethnic beneficiaries relative to White beneficiaries. Person-year logistic regression models assessing disparities included indicators for race and ethnicity, year treated as a continuous variable, age, sex, dual eligibility (both Medicare and Medicaid) status, fixed effects for state, and standard errors clustered on beneficiary identifier. To assess annual trends in disparities, models also included year × race and ethnicity interaction terms.

Our study had several limitations. First, we relied on documented diagnoses to identify beneficiaries with OUD or opioid overdose; true OUD rates may differ, and racial and ethnic disparities in identifying OUD treatment need^10^ may have resulted in conservative estimates of buprenorphine disparities. Second, dual-eligible beneficiaries could have accessed methadone treatment via their Medicaid coverage, and unobserved methadone use could have led to overestimated unmet treatment need. Third, some overdose events may be unrelated to an associated OUD, potentially overestimating treatment need. Finally, the RTI race and ethnicity variable may contain errors, especially for American Indian/Alaska Native enrollees.^7^

## Study Results

We identified 744,773 Medicare beneficiaries during the period 2015–19 with an OUD diagnosis (92.6 percent), an opioid overdose diagnosis (3.7 percent), or both (3.7 percent); 77.7 percent were White, 14.2 percent were Black, 6.3 percent were Hispanic, 1.0 percent were American Indian/Alaska Native; and 0.8 percent were Asian/Pacific Islander. Approximately 54 percent in a given calendar year were eligible on the basis of disability. See appendix exhibits A3 and A4^9^ for annual sample sizes, by Medicare eligibility category and racial and ethnic group, both overall and among those with a buprenorphine prescription fill.

### BUPRENORPHINE RECEIPT AMONG DISABILITY BENEFICIARIES

The percentage of Medicare disability beneficiaries with an OUD or opioid overdose diagnosis receiving buprenorphine increased from 2015 to 2019 for all racial and ethnic groups but remained below 25 percent for all groups in each year (exhibit 1). The increase was most pronounced for American Indian/Alaska Native beneficiaries, increasing 193 percent versus 92 percent for Black, 62 percent for Hispanic, 66 percent for Asian/Pacific Islander, and 74 percent for White beneficiaries. However, all minoritized racial and ethnic groups had lower buprenorphine receipt rates than White beneficiaries in each year. Relative to White beneficiaries, buprenorphine receipt was lowest among Black beneficiaries, at 4.9 percent in 2015 (36 percent of the rate for Whites), without substantial improvement throughout the study period (exhibit 2). The disparity improved for American Indian/Alaska Native beneficiaries (from 54 percent of the White rate in 2015 to 89 percent in 2019; *p* < 0.001), but it worsened for Hispanic beneficiaries (from 75 percent to 68 percent; *p* < 0.001) (appendix exhibits A5 and A8).^9^

Exhibits 3–6 show the state-level disparity ratios for Medicare disability beneficiaries in each of the four racial and ethnic groups relative to White beneficiaries. Nearly all states had lower buprenorphine rates for Black compared with White beneficiaries (exhibit 3), and in thirty-three states Black beneficiaries received buprenorphine at less than half the rate of their White counterparts. In five states (Alabama, Florida, Kentucky, Mississippi, and Utah), the Black-to-White disparity ratio was lower than 25 percent, whereas just four states (Alaska, Kansas, Maryland, and Vermont) and Washington, D.C., had disparity ratios of 75 percent or more. Although smaller in magnitude, we also found disparities for other racial and ethnic groups in many states. Conversely, American Indian/Alaska Native beneficiaries’ buprenorphine receipt rates exceeded those of White beneficiaries in nearly half of the states (*n* = 14) that had sufficient data (*n* = 30) (exhibit 6). State-level percentages and ratio values are shown in appendix exhibits A9 and A10.^9^

### BUPRENORPHINE RECEIPT AMONG OLDER ADULT BENEFICIARIES

Buprenorphine rates among older adults (ages sixty-five and older) increased from 2015 to 2019 for all racial and ethnic groups but remained strikingly low, below 9 percent, for all groups in each year (appendix exhibit A11).^9^ Disparities were less pronounced among older adults than among disability beneficiaries. Hispanic older adult beneficiaries had significantly lower absolute rates than their White counterparts during the period 2016–19 (for example, 5.1 percent versus 6.5 percent in 2019; *p* < 0.001) (appendix exhibit A11),^9^ whereas the disparity ratio reversed from 69 percent in 2015 to 138 percent in 2019 (*p* < 0.001) for American Indian/Alaska Native beneficiaries (appendix exhibit A12).^9^ Appendix exhibits A13–A16 show state-level disparities among older adult beneficiaries.^9^ The Black-to-White disparity ratio was lower than 50 percent in ten states, half of which were in the southeastern region, whereas no disparity was evident in twelve states primarily outside the southeastern region (ratio greater than 1.0). State-level percentages and ratio values are shown in appendix exhibits A21 and A22.^9^

## Discussion

We found wide and persistent racial and ethnic disparities in buprenorphine receipt, especially among Black Medicare disability beneficiaries, which may partly explain the increasing opioid overdose mortality among Black people during the study period.^6,11^ Wide variation across states suggests the need for state-level disparities tracking to help address barriers to access in minoritized communities. Noteworthy improvements in disparities for American Indian/Alaska Native beneficiaries, possibly a result of efforts to increase treatment access in Native communities,^12^ represent encouraging signs that targeted initiatives may reduce disparities.

Less encouraging is the persistent inequality for Black Medicare disability beneficiaries. Unobserved methadone receipt by dual-eligible beneficiaries could partially explain this disparity, as minoritized racial and ethnic groups are overrepresented among those using methadone as a result of historical segregation, which resulted in their closer proximity to methadone providers compared with White people.^13^ However, given that buprenorphine presents fewer disruptions to employment, family, and other responsibilities, it is critical that patients have equitable access to this medication. Our findings highlight the need to carefully examine the adequacy of provider systems serving minoritized communities and policies that may be contributing to these gaps.

Extreme disparity was especially prevalent among southeastern states, including states that have not expanded Medicaid (Alabama, Florida, Georgia, Mississippi, South Carolina, and Texas). Future research should test the effects of Medicaid expansion and other relevant policies on racial and ethnic disparities in the Medicare population. Conversely, there may be opportunities to learn from the few “bright spots” (for example, Alaska, Kansas, Maryland, Vermont, and Washington, D.C.) where parity or near-parity was attained. For example, Maryland undertook substantial initiatives to expand buprenorphine access in Baltimore, including the Baltimore Buprenorphine Initiative,^14^ expansion of buprenorphine in methadone opioid treatment programs, and integration of buprenorphine into formerly “drug-free”^15,16^ and syringe service^17^ programs.

The intersection of racial discrimination and structural racism with age, disability status, and other identities could partially account for differential findings by eligibility category, highlighting the need to study these populations independently. Compared with older adults, Medicare disability beneficiaries have higher prevalence of mental health disorders and are more likely to be socioeconomically disadvantaged,^18^ which likely worsens disparities.

Despite rapid overall treatment expansion, our results suggest persistent racial and ethnic disparities in buprenorphine receipt among Medicare beneficiaries; sustained, multilevel efforts are needed to address them. Policy initiatives could financially incentivize expansion of buprenorphine at sites that serve low-income and minoritized populations. Health plans should ensure network adequacy and adequate reimbursement of Medicare buprenorphine providers. Recent federal policy changes, including the elimination of required waivers for buprenorphine prescribing, could reduce disparities by broadening the provider base. Other equity-focused policies include ensuring rigorous monitoring of equity in care practices, holding health care systems accountable for observed inequities, and robust education on countering individual and institutional biases. To address structural barriers, interventions that go beyond the health care system to address upstream socioeconomic inequities across many domains (for example, housing, education, and criminal justice)^19^ are needed.

## Supplementary Material

Supplemental Material

## Figures and Tables

**EXHIBIT 1:**
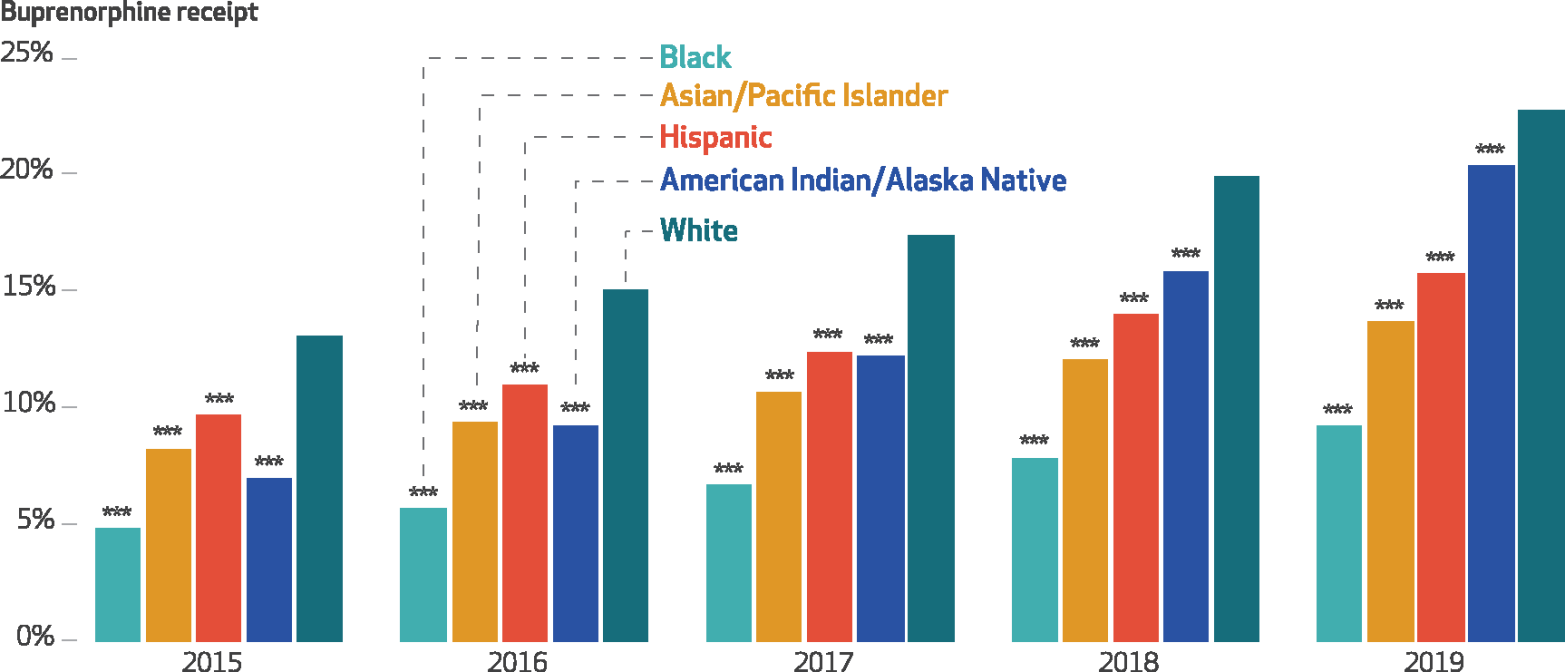
Rates of buprenorphine receipt among Medicare disability beneficiaries with opioid use disorder (OUD) or opioid overdose diagnoses in the US, by race and ethnicity, 2015–19 **SOURCE** Authors’ analysis of Medicare claims data from a 20% random sample of Part D enrollees with fee-for-service coverage, restricted to disability beneficiaries ages 18–64 with at least 1 outpatient claim with an OUD or opioid overdose diagnosis in any study calendar year, 2015–19. **NOTES** This exhibit shows the percent of beneficiaries who received buprenorphine conditional on an OUD or opioid overdose diagnosis. Within each year, rates of buprenorphine receipt for each racial and ethnic group were compared with the rate in the White group. Statistical significance was determined using logistic regression with race and ethnicity, age, sex, dual eligibility status, year treated as a continuous variable, a year × race and ethnicity interaction term, fixed effects for state, and standard errors clustered on beneficiary identifier. ***p < 0.01

**EXHIBIT 2:**
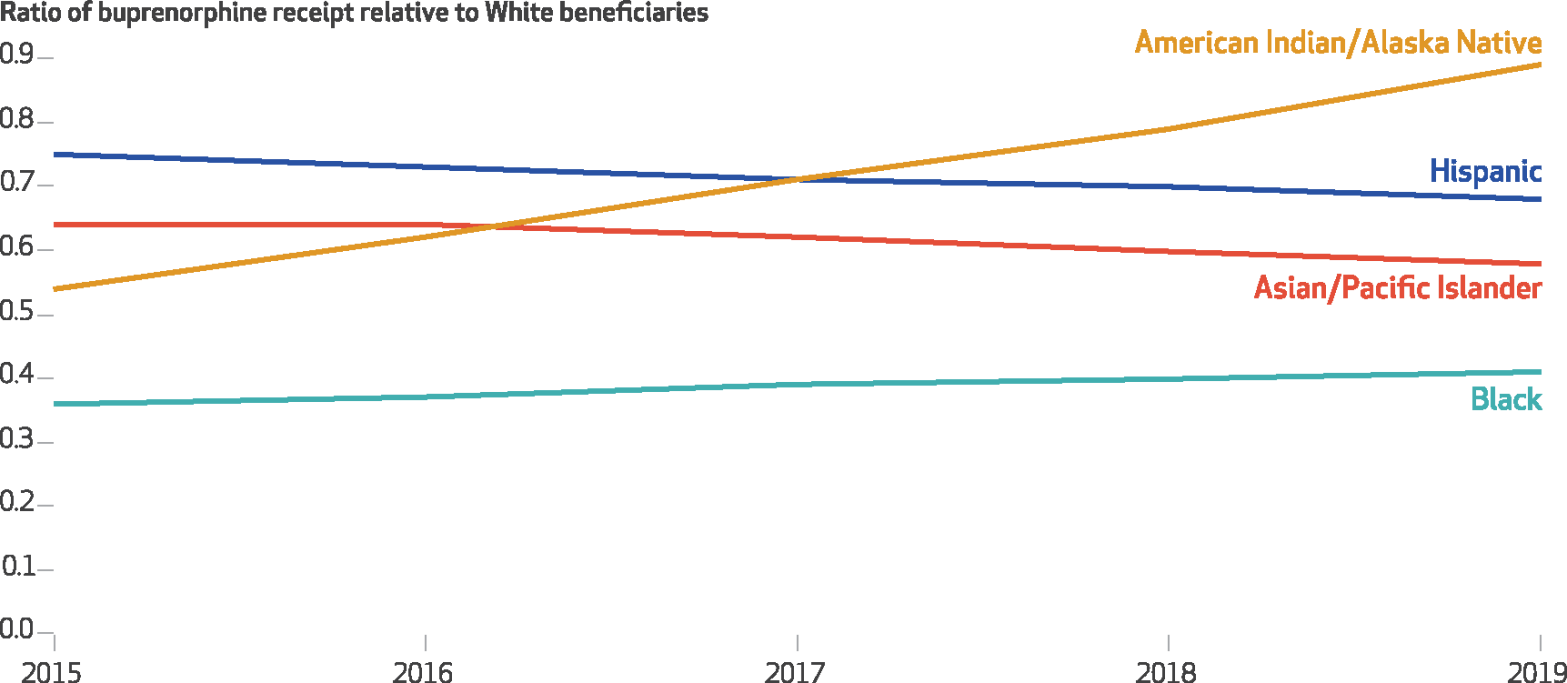
Trends in the ratio of Black, American Indian/Alaska Native, Asian/Pacific Islander, and Hispanic Medicare disability beneficiaries with opioid use disorder (OUD) or opioid overdose diagnoses receiving buprenorphine relative to their White counterparts, 2015–19 **SOURCE** Authors’ analysis of Medicare claims data from a 20% random sample of Part D enrollees with fee-for-service coverage, restricted to disability beneficiaries ages 18–64 with at least 1 outpatient claim with an OUD or opioid overdose diagnosis in any study calendar year, 2015–19. **NOTES** This exhibit shows trends in the ratio relative to White beneficiaries of receiving buprenorphine conditional on an OUD or opioid overdose diagnosis for each racial and ethnic group. A value of 1.0 indicates parity with White beneficiaries’ buprenorphine receipt. Across the study period, each additional year was associated with a significant reduction in the disparity ratio for American Indian/Alaska Native beneficiaries and a significant increase for Hispanic beneficiaries (both *p* < 0.001 in each subsequent study year). Statistical significance was determined using logistic regression with race and ethnicity, age, sex, dual eligibility status, year treated as a continuous variable, a year × race and ethnicity interaction term, fixed effects for state, and standard errors clustered on beneficiary identifier.

**EXHIBIT 3:**
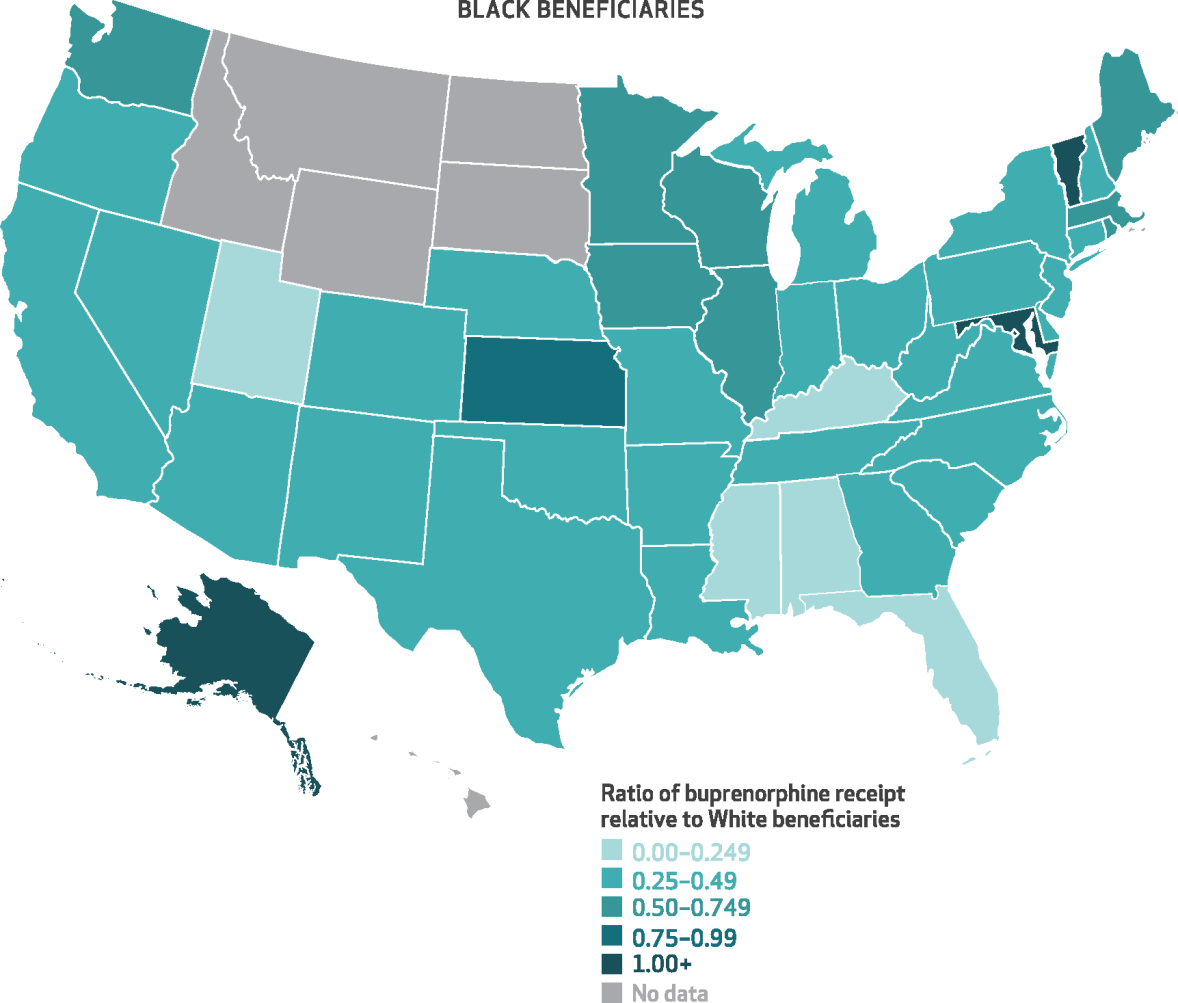
State-level ratios of percent of Black Medicare disability beneficiaries with an opioid use disorder (OUD) or opioid overdose diagnosis who received buprenorphine relative to their White counterparts, 2015–19 **SOURCE** Authors’ analysis of Medicare claims data from a 20% random sample of Part D enrollees with fee-for-service coverage, restricted to disability beneficiaries ages 18–64 with at least 1 outpatient claim with an OUD or opioid overdose diagnosis in any study calendar year, 2015–19. **NOTES** This exhibit shows a color-coded US map with each state shaded in according to the average percent of Black Medicare disability beneficiaries who received buprenorphine conditional on an OUD or opioid overdose diagnosis relative to that of their White counterparts. Darker shades of teal indicate smaller ratios and greater disparities, whereas lighter shading indicates more equal ratios of buprenorphine receipt in a given state. “No data” applies to states in which there were fewer than 50 total Black beneficiaries across the study period.

**EXHIBIT 4:**
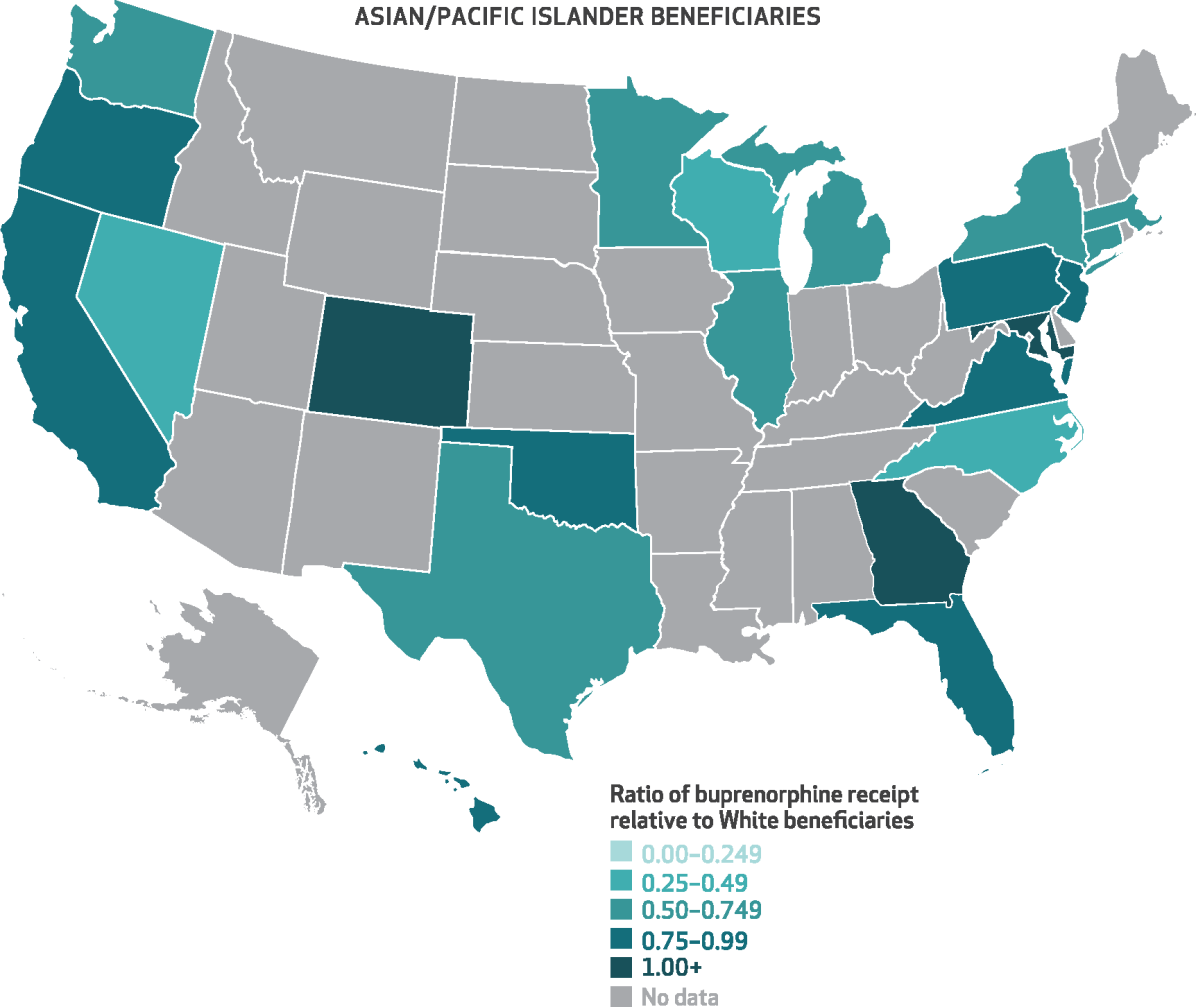
State-level ratios of percent of Asian/Pacific Islander Medicare disability beneficiaries with an opioid use disorder (OUD) or opioid overdose diagnosis who received buprenorphine relative to their White counterparts, 2015–19 **SOURCE** Authors’ analysis of Medicare claims data from a 20% random sample of Part D enrollees with fee-for-service coverage, restricted to disability beneficiaries ages 18–64 with at least 1 outpatient claim with an OUD or opioid overdose diagnosis in any study calendar year, 2015–19. **NOTES** This exhibit shows a color-coded US map with each state shaded in according to the average percent of Asian/Pacific Islander disability beneficiaries who received buprenorphine conditional on an OUD or opioid overdose diagnosis relative to that of their White counterparts. Darker shades of teal indicate smaller ratios and greater disparities, whereas lighter shading indicates more equal ratios of buprenorphine receipt in a given state. “No data” applies to states in which there were fewer than 50 total Asian/Pacific Islander beneficiaries across the study period.

**EXHIBIT 5:**
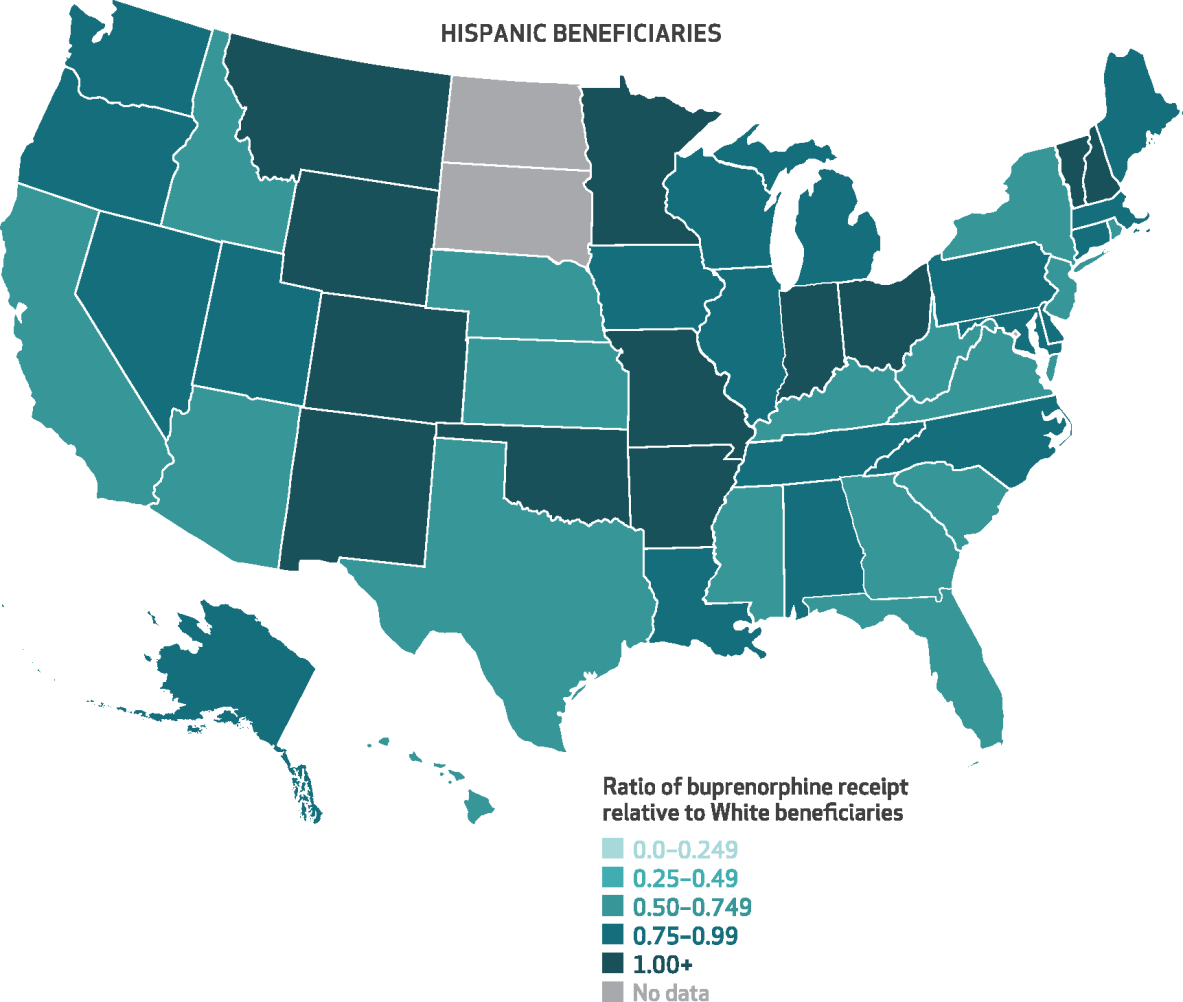
State-level ratios of percent of Hispanic Medicare disability beneficiaries with an opioid use disorder (OUD) or opioid overdose diagnosis who received buprenorphine relative to their White counterparts, 2015–19 **SOURCE** Authors’ analysis of Medicare claims data from a 20% random sample of Part D enrollees with fee-for-service coverage, restricted to disability beneficiaries ages 18–64 with at least 1 outpatient claim with an OUD or opioid overdose diagnosis in any study calendar year, 2015–19. **NOTES** This exhibit shows a color-coded US map with each state shaded in according to the average percent of Hispanic disability beneficiaries who received buprenorphine conditional on an OUD or opioid overdose diagnosis relative to that of their White counterparts. Darker shades of teal indicate smaller ratios and greater disparities, whereas lighter shading indicates more equal ratios of buprenorphine receipt in a given state. “No data” applies to states in which there were fewer than 50 total Hispanic beneficiaries across the study period.

**EXHIBIT 6:**
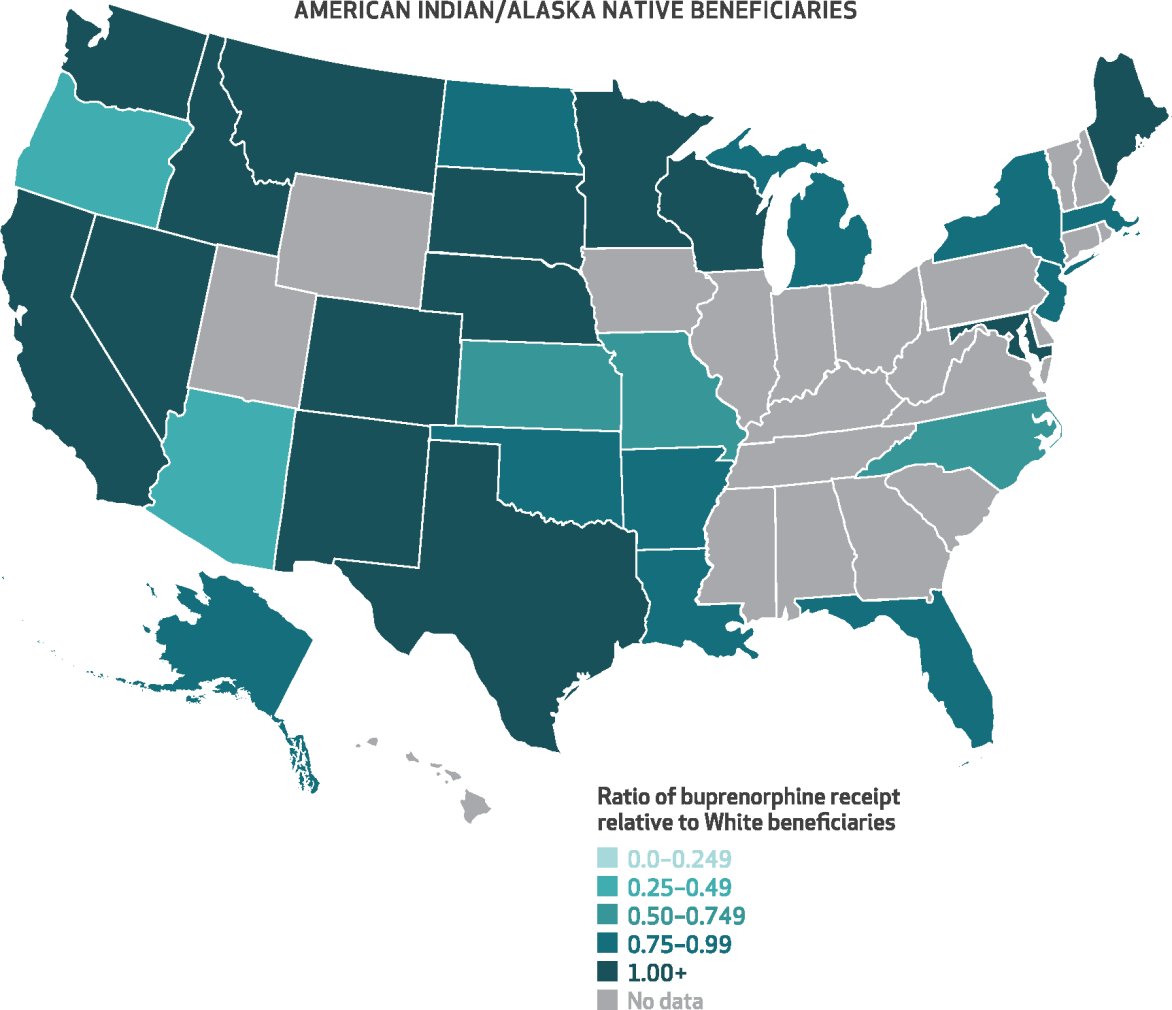
State-level ratios of percent of American Indian/Alaska Native Medicare disability beneficiaries with an opioid use disorder (OUD) or opioid overdose diagnosis who received buprenorphine relative to their White counterparts, 2015–19 **SOURCE** Authors’ analysis of Medicare claims data from a 20% random sample of Part D enrollees with fee-for-service coverage, restricted to disability beneficiaries ages 18–64 with at least 1 outpatient claim with an OUD or opioid overdose diagnosis in any study calendar year, 2015–19. **NOTES** This exhibit shows a color-coded US map with each state shaded in according to the average percent of American Indian/Alaska Native disability beneficiaries who received buprenorphine conditional on an OUD or opioid overdose diagnosis relative to that of their White counterparts. Darker shades of teal indicate smaller ratios and greater disparities, whereas lighter shading indicates more equal ratios of buprenorphine receipt in a given state. “No data” applies to states in which there were fewer than 50 total American Indian/Alaska Native beneficiaries across the study period.
