# Supplementary material for: Racial And Ethnic Disparities In Buprenorphine Receipt Among Medicare Beneficiaries, 2015–19
Source: Health Aff (Millwood). Author manuscript; Available in PMC 2024 Mar 4. (PMC10910625; doi:10.1377/hlthaff.2023.00205)
Supplement: Supplemental Material [file NIHMS1967304-supplement-Supplemental_Material.pdf]

APPENDIX

Appendix Exhibit A1. Generic drug name search terms and NDCs used to identify all buprenorphine formulations for opioid use disorder in Medicare Part D claims linked to First DataBank MedKnowledge database

Include NDC if generic drug name contains: “BUPRENORPHINE”
Exclude NDC if brand name contains: “BELBUCA”, “BUPRENEX”, “BUTRANS”, or “BUPRENORPHINE 0.3 MG/ML CRPJCT”

Note: NDC = National Drug Code; Records were manually reviewed to ensure buprenorphine formulations not indicated for OUD were not captured.

Appendix Exhibit A2. ICD-9-CM and ICD-10-CM codes used to identify opioid use disorder and opioid overdose

**Opioid Type**	**ICD-9-CM Codes**	**ICD-10-CM Codes**
Opioid use disorder	304.00, 304.01, 304.02, 304.03, 305.50, 305.51, 305.52, 305.53	F11.10, F11.120, F11.129, F11.20, F11.21, F11.220, F11.221, F11.222, F11.229, F11.23, F11.24, F11.250, F11.251, F11.259, F11.281, F11.282, F11.288, F11.29, F11.90
Opioid overdose	965.00, 965.01, 965.02, 965.09, 970.1, E85.00, E85.01, E85.02	T40.0, T40.1, T40.2, T40.3, T40.4, T40.6

Appendix Exhibit A3: Medicare beneficiaries with OUD or opioid-involved overdose, by eligibility category and racial/ethnic group, annually and for de-duplicated 2015-2019

	**2015**		**2016**		**2017**		**2018**		**2019**		**2015-2019**	
	**N**	**%**	**N**	**%**	**N**	**%**	**N**	**%**	**N**	**%**	**N**	**%**
**Overall**	199639	100.0	248547	100.0	259796	100.0	272474	100.0	272880	100.0	744773	100.0
**Disability (18-64)**												
Total	132069	66.2	153587	61.8	152764	58.8	149578	54.9	138352	50.7	400818	53.8
White	99421	75.3	114699	74.7	114501	75	112133	75	103483	74.8	295800	73.8
Black	21344	16.2	25474	16.6	24693	16.2	23809	15.9	21512	15.5	68414	17.1
Hispanic	9100	6.9	10762	7	10823	7.1	10804	7.2	10606	7.7	29103	7.3
Asian	693	0.5	783	0.5	848	0.6	875	0.6	930	0.7	2469	0.6
American Indian	1511	1.1	1869	1.2	1899	1.2	1957	1.3	1821	1.3	5032	1.3
**Older adults (65+)**												
Total	67570	33.8	94960	38.2	107032	41.2	122896	45.1	134528	49.3	343955	46.2
White	55133	81.6	77592	81.7	87758	82	101685	82.7	111856	83.1	283193	82.3
Black	7893	11.7	10927	11.5	12038	11.2	13025	10.6	13677	10.2	37018	10.8
Hispanic	3430	5.1	4817	5.1	5476	5.1	6067	4.9	6701	5	17696	5.1
Asian	564	0.8	843	0.9	979	0.9	1144	0.9	1258	0.9	3406	1
American Indian	550	0.8	781	0.8	781	0.7	975	0.8	1036	0.8	2642	0.8

SOURCE: Authors’ analysis of Medicare claims data from a 20% random sample of Part D enrollees with fee-for-service coverage, restricted to disability beneficiaries aged 18-64 years with an OUD diagnosis or opioid overdose in any study calendar year (2015-2019).

Appendix Exhibit A4: Medicare beneficiaries with OUD or opioid overdose with filled buprenorphine prescriptions, by eligibility category and racial/ethnic group, annually and for de-duplicated 2015-2019

	2015		2016		2017		2018		2019		2015-2019	
	**N**	**%**	**N**	**%**	**N**	**%**	**N**	**%**	**N**	**%**	**N**	**%**
**Overall**	19316	100.0	21573	100.0	26401	100.0	31781	100.0	36812	100.0	69743	100.0
**Disability (18-64)**												
Total	17251	89.3	18861	87.4	22277	84.4	25595	80.5	27967	76.0	55059	78.9
White	14805	85.8	16218	86	19163	86	21878	85.5	23743	84.9	46393	84.3
Black	1233	7.1	1285	6.8	1543	6.9	1828	7.1	2062	7.4	4446	8.1
Hispanic	1012	5.9	1134	6	1269	5.7	1477	5.8	1669	6	3329	6
Asian	73	0.4	65	0.3	78	0.4	111	0.4	124	0.4	252	0.5
American Indian	128	0.7	159	0.8	224	1	301	1.2	369	1.3	639	1.2
**Older adult (65+)**												
Total	2065	10.7	2712	12.6	4124	15.6	6186	19.5	8845	24.0	14684	21.1
White	1686	81.6	2233	82.3	3389	82.2	5187	83.9	7450	84.2	12224	83.2
Black	273	13.2	332	12.2	502	12.2	652	10.5	885	10	1612	11
Hispanic	85	4.1	110	4.1	169	4.1	242	3.9	344	3.9	591	4
Asian	10	0.5	18	0.7	31	0.8	46	0.7	73	0.8	112	0.8
American Indian	11	0.5	19	0.7	33	0.8	59	1	93	1.1	145	1

SOURCE: Authors' analysis of Medicare claims data from a 20% random sample of Part D enrollees with fee-for-service coverage, restricted to disability beneficiaries aged 18-64 years with an OUD diagnosis or opioid overdose in any study calendar year (2015-2019) with a filled buprenorphine prescription in the same calendar year.

Appendix Exhibit A5. Logistic regression of buprenorphine receipt on year and race/ethnicity among Medicare disability beneficiaries with OUD or opioid overdose, 2015-2019 (n = 400,818)

**Variable**	**aOR**	**95% CI**	**p-value**
Year	1.19	1.19-1.20	<.001
Race/ethnicity			
White	REF	REF	REF
American Indian/Alaska Native	0.68	0.57-0.81	<.001
Asian/Pacific Islander	0.62	0.49-0.79	<.001
Black	0.36	0.34-0.39	<.001
Hispanic	0.72	0.68-0.77	<.001
Age	0.96	0.96-0.96	<.001
Male sex	1.19	1.17-1.22	<.001
Enrolled in Medicaid	1.08	1.05-1.11	<.001
Interactions			
Year*White	REF	REF	REF
Year*American Indian/Alaska Native	1.15	1.10-1.21	<.001
Year*Asian/Pacific Islander	0.97	0.89-1.05	.429
Year* Black	1.00	0.98-1.02	.943
Year*Hispanic	0.97	0.95-0.99	.002

SOURCE: Authors' analysis of Medicare claims data from a 20% random sample of Part D enrollees with fee-for-service coverage, restricted to disability beneficiaries aged 18-64 years with at least 1 claim with an OUD or opioid overdose diagnosis in any study calendar year (2015-2019).

NOTES: Estimates are from an individual-level logistic regression model with standard errors clustered on beneficiary ID and fixed effect for state. aOR = adjusted odds ratio; CI = confidence interval

Appendix Exhibit A6. Logistic regression of buprenorphine receipt on year and race/ethnicity among Medicare disability beneficiaries with OUD or opioid overdose, 2015-2019, sensitivity analysis using CCW OUD definition (N = 219,790)

**Variable**	**aOR**	**95% CI**	**p-value**
Year	1.28	1.27-1.29	<.001
Race/ethnicity			
White			REF
American Indian/Alaska Native	0.66	0.52-0.83	<.001
Asian/Pacific Islander	0.68	0.50-0.94	.018
Black	0.50	0.46-0.53	<.001
Hispanic	0.82	0.75-0.89	<.001
Age	0.97	0.97-0.97	<.001
Male sex	1.11	1.08-1.15	<.001
Enrolled in Medicaid	1.10	1.05-1.14	<.001
Interactions			
Year*White	REF	REF	REF
Year*American Indian/Alaska Native	1.14	1.06-1.24	<.001
Year*Asian/Pacific Islander	0.99	0.88-1.12	.920
Year* Black	0.98	0.95-1.00	.070
Year*Hispanic	0.97	0.94-1.00	.025

SOURCE: Authors' analysis of Medicare claims data from a 20% random sample of Part D enrollees with fee-for-service coverage, restricted to disability beneficiaries aged 18-64 years with an OUD diagnosis or opioid overdose based on the Chronic Condition Data Warehouse definition of opioid use disorder (1 inpatient *or* 2 outpatient claims with an OUD or opioid overdose diagnosis) in any study year (2015-2019).

NOTES: Estimates are from an individual-level logistic regression model with standard errors clustered on beneficiary ID and fixed effect for state. aOR = adjusted odds ratio; CI = confidence interval

Appendix Exhibit A7. Logistic regression of buprenorphine receipt on year and race/ethnicity among Medicare disability beneficiaries with OUD or opioid overdose, sensitivity analysis excluding beneficiaries dually enrolled in Medicaid, 2015-2019 (N = 177,550)

**Variable**	**aOR**	**95% CI**	**p-value**
Year	1.17	1.15-1.18	<.001
Race/ethnicity			
White	REF	REF	REF
American Indian/Alaska Native	0.77	0.51-1.15	.196
Asian/Pacific Islander	0.70	0.43-1.12	.138
Black	0.39	0.34-0.46	<.001
Hispanic	0.79	0.68-0.93	.004
Age	0.96	0.96-0.96	<.001
Male sex	1.39	1.33-1.46	<.001
Interactions			
Year*White	REF	REF	REF
Year*American Indian/Alaska Native	1.15	1.01-1.31	.030
Year*Asian/Pacific Islander	0.88	0.75-1.05	.155
Year* Black	0.99	0.94-1.04	.732
Year*Hispanic	0.98	0.93-1.03	.420

SOURCE: Authors' analysis of Medicare claims data from a 20% random sample of Part D enrollees with fee-for-service coverage, restricted to non-dual eligible disability beneficiaries aged 18-64 years with at least 1 claim with an OUD or opioid overdose diagnosis in any study calendar year (2015-2019).

NOTES: Estimates are from an individual-level logistic regression model with standard errors clustered on beneficiary ID and fixed effect for state. aOR = adjusted odds ratio; CI = confidence interval

Appendix Exhibit A8. Adjusted ratios of percentage of disability beneficiaries with OUD or opioid overdose who received buprenorphine relative to White disability beneficiaries, 2015-2019 (n= 400,818)

**Variable**	**Adjusted Ratio**	**95% CI**
2015		
White	REF	REF
American Indian/Alaska Native	0.54	0.45-0.65
Asian/Pacific Islander	0.64	0.50-0.81
Black	0.36	0.34-0.38
Hispanic	0.75	0.70-0.80
2016		
White	REF	REF
American Indian/Alaska Native	0.62	0.54-0.71
Asian/Pacific Islander	0.64	0.52-0.79
Black	0.37	0.36-0.39
Hispanic	0.73	0.69-0.77
2017		
White	REF	REF
American Indian/Alaska Native	0.71	0.63-0.80
Asian/Pacific Islander	0.62	0.51-0.75
Black	0.39	0.37-0.41
Hispanic	0.71	0.68-0.75
2018		
White	REF	REF
American Indian/Alaska Native	0.79	0.71-0.87
Asian/Pacific Islander	0.60	0.50-0.72
Black	0.40	0.38-0.42
Hispanic	0.70	0.67-0.74
2019		
White	REF	REF
American Indian/Alaska Native	0.89	0.81-0.98
Asian/Pacific Islander	0.58	0.49-0.69
Black	0.41	0.39-0.42
Hispanic	0.68	0.65-0.71

SOURCE: Authors’ analysis of Medicare claims data from a 20% random sample of Part D enrollees with fee-for-service coverage, restricted to disability beneficiaries aged 18-64 years with at least 1 claim with an OUD or opioid overdose diagnosis in any study calendar year (2015-2019).

NOTES: Adjusted ratios and CIs use predicted probabilities within each year and racial/ethnic group from an individual-level logistic regression model shown in Appendix Exhibit A5. aOR = adjusted odds ratio; CI = confidence interval

Appendix Exhibit A9: Unadjusted state percentage of disability beneficiaries with OUD or opioid overdose who received buprenorphine, 2015-2019

State	White %	Black %	Hispanic %	Asian/Pacific Islander %	American Indian/Alaska Native %
National – All States	17.6	6.8	12.5	8.2	11.2
Alabama	22.0	4.9	16.6	NR	NR
Alaska	14.3	15.7	NR	NR	14.1
Arizona	7.8	3.3	5.8	NR	3.0
Arkansas	6.9	2.1	7.3	NR	NR
California	12.6	3.6	7.3	9.6	16.4
Colorado	9.0	4.0	9.5	NR	NR
Connecticut	19.4	5.6	18.8	NR	NR
Delaware	11.2	4.7	8.7	NR	NR
District of Columbia	12.5	18.3	NR	NR	NR
Florida	14.6	3.1	9.6	11.6	14.3
Georgia	11.4	3.3	6.4	23.5	NR
Hawaii	13.0	NR	NR	10.5	NR
Idaho	13.8	NR	7.9	NR	15.4
Illinois	11.9	7.2	11.7	NR	NR
Indiana	20.7	9.8	22.8	NR	NR
Iowa	4.9	3.5	NR	NR	NR
Kansas	6.7	5.7	NR	NR	NR
Kentucky	24.1	4.7	16.8	NR	NR
Louisiana	14.5	3.9	11.3	NR	NR
Maine	34.9	19.8	32.5	NR	38.9
Maryland	15.4	15.6	11.6	15.9	NR
Massachusetts	32.9	21.9	30.3	22.3	31.3
Michigan	18.5	7.0	16.7	12.7	17.5
Minnesota	9.7	5.7	9.9	NR	13.8
Mississippi	14.2	2.8	8.5	NR	NR
Missouri	12.8	5.1	19.0	NR	NR
Montana	13.3	NR	23.1	NR	19.6
Nebraska	9.8	NR	NR	NR	NR
Nevada	7.1	2.0	5.4	NR	12.0
New Hampshire	29.5	13.3	32.7	NR	NR
New Jersey	16.7	5.8	9.0	13.7	NR
New Mexico	11.6	NR	18.6	NR	18.5
New York	21.7	7.6	13.0	10.8	20.6
North Carolina	18.5	7.6	14.5	NR	11.7
North Dakota	7.3	NR	NR	NR	NR
Ohio	24.9	9.9	25.5	NR	NR
Oklahoma	9.1	3.0	9.5	NR	8.1
Oregon	14.7	4.2	13.3	13.0	NR
Pennsylvania	17.8	8.4	14.8	14.9	NR
Rhode Island	34.1	21.6	25.4	NR	NR
South Carolina	17.2	6.0	10.7	NR	NR
South Dakota	10.5	NR	NR	NR	12.6
Tennessee	14.9	4.1	14.7	NR	NR
Texas	11.1	3.6	5.7	8.0	12.4
Utah	17.0	NR	15.8	NR	NR

Vermont	28.4	33.3	41.2	NR	NR
Virginia	20.5	6.8	13.9	NR	NR
Washington	16.3	8.3	13.0	9.8	19.6
West Virginia	28.8	10.9	19.6	NR	NR
Wisconsin	12.4	7.8	10.0	NR	15.5
Wyoming	17.3	NR	25.0	NR	NR

SOURCE: Authors' analysis of Medicare claims data from a 20% random sample of Part D enrollees with fee-for-service coverage, restricted to disability beneficiaries aged 18-64 years with at least 1 claim with an OUD or opioid overdose diagnosis in any study calendar year (2015-2019).

NOTES: Racial/ethnic groups with <50 total individuals in the state across the study period are not reported (NR) due to small cell sizes.

Appendix Exhibit A10: Unadjusted state ratios of percentage of beneficiaries with OUD or opioid overdose who received buprenorphine relative to White disability beneficiaries, 2015-2019

State	Black	Hispanic	Asian/Pacific Islander	American Indian/Alaska Native
	Ratio (95% CI)	Ratio (95% CI)	Ratio (95% CI)	Ratio (95% CI)
National – All States	0.39 (0.38-0.40)	0.72 (0.70-0.73)	0.62 (0.57-0.68)	0.74 (0.70-0.78)
Alabama	0.22 (0.19-0.26)	0.75 (0.53-1.07)	NR	NR
Alaska	1.10 (0.70-1.74)	0.83 (0.41-1.68)	NR	0.99 (0.73-1.34)
Arizona	0.42 (0.26-0.66)	0.74 (0.58-0.95)	NR	0.38 (0.23-0.64)
Arkansas	0.30 (0.20-0.44)	1.06 (0.60-1.89)	NR	0.90 (0.41-1.95)
California	0.29 (0.25-0.33)	0.58 (0.54-0.63)	0.77 (0.63-0.93)	1.30 (1.11-1.53)
Colorado	0.45 (0.29-0.68)	1.05 (0.90-1.23)	1.11 (0.48-2.56)	1.44 (0.80-2.58)
Connecticut	0.29 (0.23-0.37)	0.97 (0.87-1.09)	0.69 (0.36-1.31)	NR
Delaware	0.42 (0.34-0.53)	0.78 (0.52-1.17)	NR	NR
District of Columbia	1.47 (0.79-2.72)	NR	NR	NR
Florida	0.21 (0.18-0.25)	0.66 (0.59-0.74)	0.80 (0.50-1.29)	0.98 (0.57-1.70)
Georgia	0.29 (0.24-0.34)	0.57 (0.36-0.88)	2.07 (1.26-3.41)	NR
Hawaii	NR	0.28 (0.09-0.86)	0.81 (0.53-1.24)	NR
Idaho	NR	0.57 (0.37-0.90)	NR	1.11 (0.68-1.82)
Illinois	0.60 (0.54-0.67)	0.98 (0.84-1.16)	0.52 (0.20-1.34)	NR
Indiana	0.47 (0.41-0.54)	1.10 (0.90-1.33)	NR	NR
Iowa	0.71 (0.39-1.29)	0.93 (0.39-2.21)	NR	NR
Kansas	0.85 (0.58-1.25)	0.51 (0.26-1.02)	NR	0.62 (0.20-1.88)
Kentucky	0.19 (0.15-0.25)	0.70 (0.52-0.95)	NR	NR
Louisiana	0.27 (0.23-0.31)	0.78 (0.58-1.06)	NR	0.86 (0.43-1.73)
Maine	0.57 (0.37-0.87)	0.93 (0.68-1.28)	NR	1.11 (0.86-1.44)
Maryland	1.01 (0.95-1.08)	0.75 (0.57-1.00)	1.03 (0.62-1.69)	1.25 (0.71-2.18)
Massachusetts	0.67 (0.62-0.72)	0.92 (0.88-0.97)	0.68 (0.51-0.91)	0.95 (0.67-1.36)
Michigan	0.38 (0.35-0.41)	0.90 (0.78-1.04)	0.69 (0.42-1.12)	0.94 (0.73-1.22)
Minnesota	0.59 (0.49-0.70)	1.02 (0.74-1.42)	0.63 (0.30-1.29)	1.42 (1.15-1.75)
Mississippi	0.20 (0.17-0.24)	0.60 (0.34-1.05)	NR	NR
Missouri	0.40 (0.32-0.49)	1.49 (1.15-1.92)	NR	0.74 (0.35-1.60)
Montana	NR	1.73 (1.04-2.88)	NR	1.47 (1.14-1.89)
Nebraska	0.49 (0.26-0.91)	0.70 (0.35-1.39)	NR	1.05 (0.49-2.27)
Nevada	0.28 (0.19-0.41)	0.76 (0.55-1.05)	0.30 (0.08-1.19)	1.69 (1.06-2.72)
New Hampshire	0.45 (0.26-0.78)	1.11 (0.91-1.36)	NR	NR
New Jersey	0.35 (0.32-0.38)	0.54 (0.49-0.60)	0.82 (0.60-1.12)	0.95 (0.52-1.73)
New Mexico	0.31 (0.12-0.81)	1.61 (1.40-1.85)	NR	1.60 (1.20-2.12)
New York	0.35 (0.32-0.39)	0.60 (0.55-0.66)	0.50 (0.33-0.74)	0.95 (0.68-1.31)
North Carolina	0.41 (0.38-0.45)	0.78 (0.65-0.95)	0.39 (0.17-0.91)	0.63 (0.51-0.77)
North Dakota	NR	NR	NR	0.92 (0.48-1.74)
Ohio	0.40 (0.35-0.45)	1.03 (0.89-1.19)	NR	NR
Oklahoma	0.33 (0.26-0.43)	1.04 (0.80-1.35)	0.77 (0.30-1.99)	0.89 (0.77-1.03)
Oregon	0.28 (0.16-0.51)	0.90 (0.69-1.18)	0.89 (0.53-1.48)	0.42 (0.23-0.76)
Pennsylvania	0.47 (0.43-0.52)	0.83 (0.75-0.93)	0.84 (0.56-1.26)	NR
Rhode Island	0.63 (0.48-0.83)	0.74 (0.62-0.09)	NR	NR
South Carolina	0.35 (0.29-0.43)	0.62 (0.37-1.04)	NR	NR
South Dakota	NR	NR	NR	1.20 (0.75-1.92)
Tennessee	0.27 (0.22-0.34)	0.99 (0.74-1.31)	NR	NR
Texas	0.32 (0.28-0.36)	0.52 (0.46-0.58)	0.72 (0.42-1.21)	1.11 (0.74-1.66)
Utah	0.08 (0.01-0.57)	0.93 (0.72-1.20)	NR	NR
Vermont	1.17 (0.81-1.70)	1.45 (1.04-2.02)	NR	NR

Virginia	0.33 (0.29-0.38)	0.68 (0.50-0.93)	0.78 (0.41-1.48)	NR
Washington	0.51 (0.41-0.63)	0.80 (0.67-0.94)	0.60 (0.40-0.90)	1.20 (1.01-1.43)
West Virginia	0.38 (0.26-0.55)	0.68 (0.39-1.19)	NR	NR
Wisconsin	0.63 (0.52-0.77)	0.81 (0.61-1.06)	0.38 (0.13-1.15)	1.25 (0.93-1.68)
Wyoming	NR	1.45 (0.91-2.29)	NR	NR

SOURCE: Authors' analysis of Medicare claims data from a 20% random sample of Part D enrollees with fee-for-service coverage, restricted to disability beneficiaries aged 18-64 years with at least 1 claim with an OUD or opioid overdose diagnosis in any study calendar year (2015-2019).

NOTES: Racial/ethnic groups with <50 total individuals in the state across the study period are not reported (NR) due to small cell sizes. Ratios are calculated as the percentage of individuals in each minoritized racial/ethnic group who received buprenorphine divided by the percentage of white individuals who received buprenorphine across the study period (2015-2019). CI= Confidence Interval

Appendix Exhibit A11: National rates of buprenorphine receipt among Medicare older adult beneficiaries with opioid use disorder or opioid overdose, by race/ethnicity

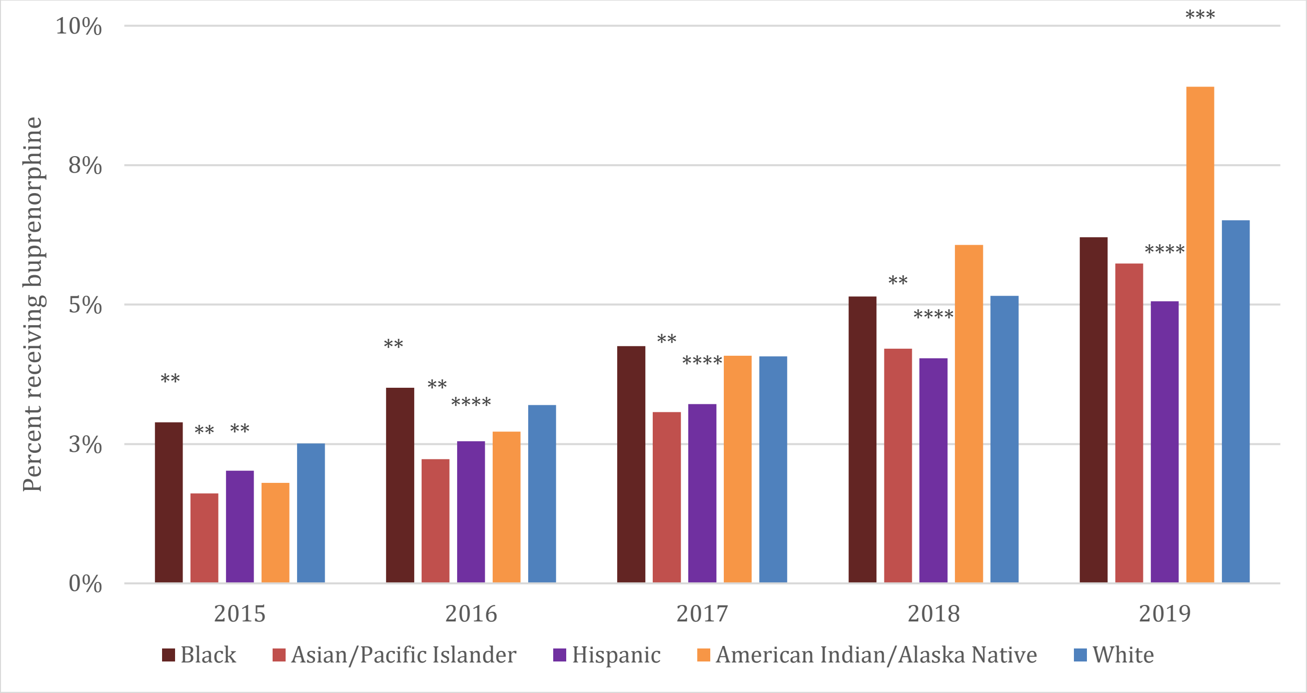

SOURCE: Authors analysis of Medicare claims data from a 20% random sample of Part D enrollees with fee-for-service coverage, restricted to older adult beneficiaries aged 65+ years with at least 1 outpatient claim with an OUD diagnosis or opioid overdose in any study year (2015-2019).

NOTES: Appendix Exhibit A11 shows the percentage of beneficiaries who received buprenorphine conditional on OUD diagnosis or opioid overdose. In each year, Hispanic beneficiaries had statistically lower rates of buprenorphine receipt compared to Whites (all $p < .05$). Notably, Black beneficiaries' receipt of buprenorphine only differed significantly from Whites in 2015 and 2016. Statistical significance was determined using logistic regression with race/ethnicity, year, a race/ethnicity by year interaction, age, sex, dual eligibility status, fixed-effect for state, and standard errors clustered on beneficiary ID.
* $p < 0.1$, ** $p < 0.05$, *** $p < 0.01$, **** $p < 0.001$

Appendix Exhibit A12. National trends in the ratio of receiving buprenorphine relative to White older adult Medicare enrollees with OUD diagnosis or opioid overdose, 2015-2019

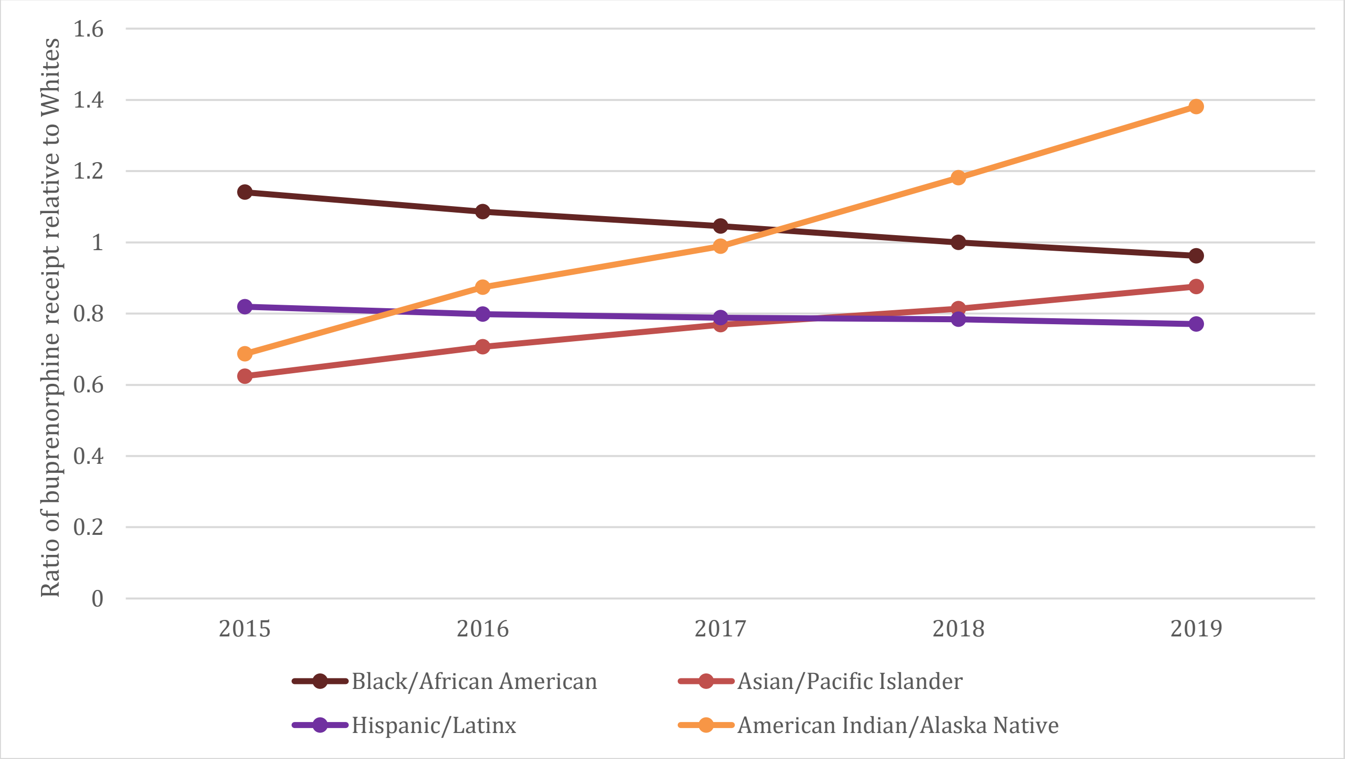

SOURCE: Authors analysis of Medicare claims data from a 20% random sample of Part D enrollees with fee-for-service coverage, restricted to older adult beneficiaries aged 65+ years with at least 1 outpatient claim with an OUD diagnosis or opioid overdose in any study year (2015-2019).

NOTES: Appendix Exhibit A12 shows trends in the ratio relative to White beneficiaries of receiving buprenorphine conditional on OUD diagnosis or opioid overdose. Values that are close to or above 1 are at parity (or above parity) with White buprenorphine receipt. Across the study period, the disparity ratio for Black beneficiaries reduced significantly to be nearly the same rate as White beneficiaries by 2019 ($p<0.01$), while the disparity ratio reversed for American Indian/Alaska Native beneficiaries, rising from 69% of Whites in 2015 to 138% of White beneficiaries by 2019 ($p<0.05$). Statistical significance was determined using logistic regression with race/ethnicity, year, a race/ethnicity by year interaction, age, sex, dual eligibility status, fixed-effect for state, and standard errors clustered on beneficiary ID.

Appendix Exhibit A13. State-level ratio of percentage of Black patients with OUD receiving buprenorphine to White patients with OUD receiving buprenorphine, 65+, 2015-2019

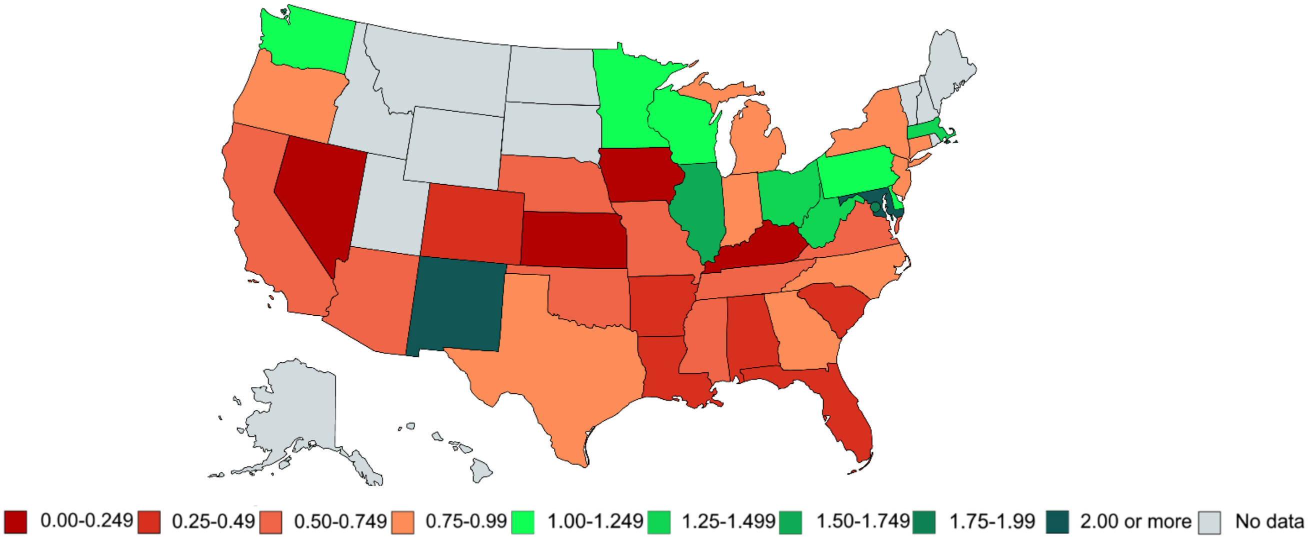

SOURCE: Authors' analysis of Medicare claims data from a 20% random sample of Part D enrollees with fee-for-service coverage, restricted to older adult beneficiaries aged 65+ years with at least 1 outpatient claim with an OUD diagnosis or opioid overdose in any study year (2015-2019).

NOTES: Appendix exhibit A13 shows color-coded map of the United States with each state shaded in based on the average percent of older adult Black beneficiaries who received buprenorphine conditional on an OUD or overdose diagnosis between 2015-2019 relative to that of their White counterparts. Darker shades of red indicate smaller ratios and greater disparities, while states shaded in green indicate more equal ratios of buprenorphine receipt in a given state. States where the racial/ethnic group has <50 total individuals across the study period are shaded gray and indicated as 'no data' on maps.

Appendix Exhibit A14. State-level ratio of percentage of Hispanic patients with OUD receiving buprenorphine to White patients with OUD receiving buprenorphine, 65+, 2015-2019

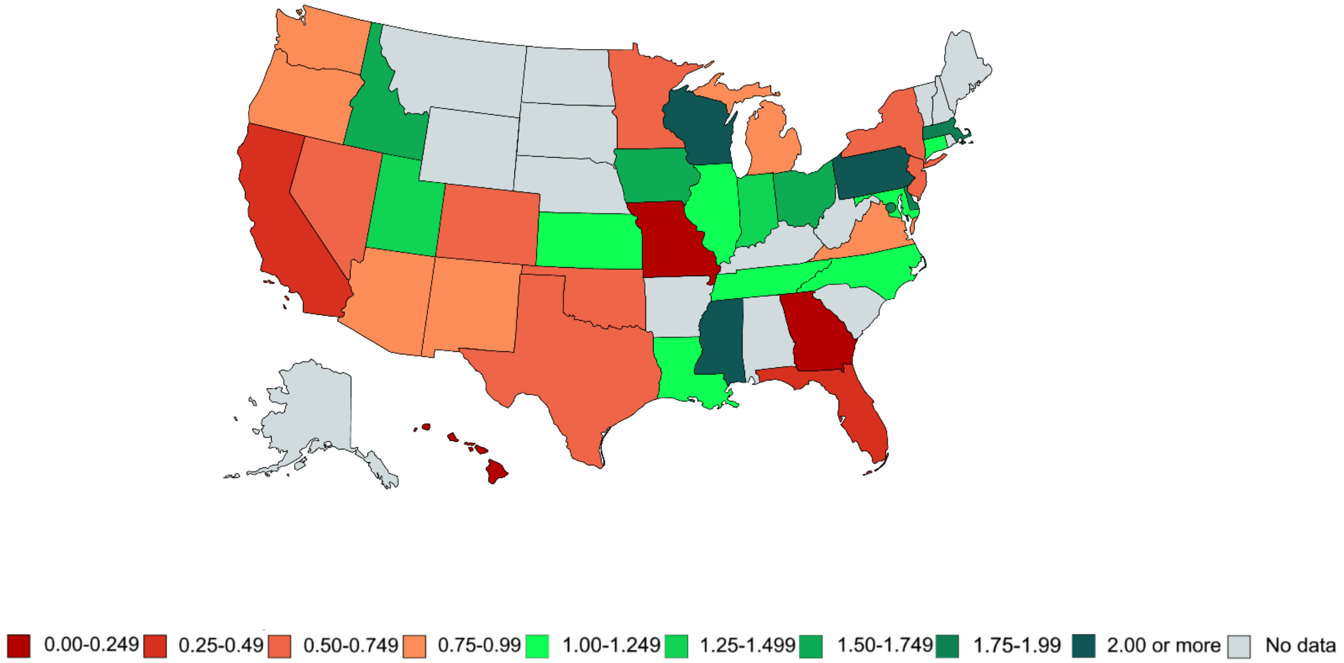

SOURCE: Authors' analysis of Medicare claims data from a 20% random sample of Part D enrollees with fee-for-service coverage, restricted to older adult beneficiaries aged 65+ years with at least 1 outpatient claim with an OUD diagnosis or opioid overdose in any study year (2015-2019).

NOTES: Appendix exhibit A14 shows color-coded map of the United States with each state shaded in based on the average percent of older adult Hispanic beneficiaries who received buprenorphine conditional on an OUD or overdose diagnosis between 2015-2019 relative to that of their White counterparts. Darker shades of red indicate smaller ratios and greater disparities, while states shaded in green indicate more equal ratios of buprenorphine receipt in a given state. States where the racial/ethnic group has <50 total individuals across the study period are shaded gray and indicated as 'no data' on maps.

Appendix Exhibit A15. State-level ratio of percentage of American Indian/Alaska Native patients with OUD receiving buprenorphine to White patients with OUD receiving buprenorphine, 65+, 2015-2019

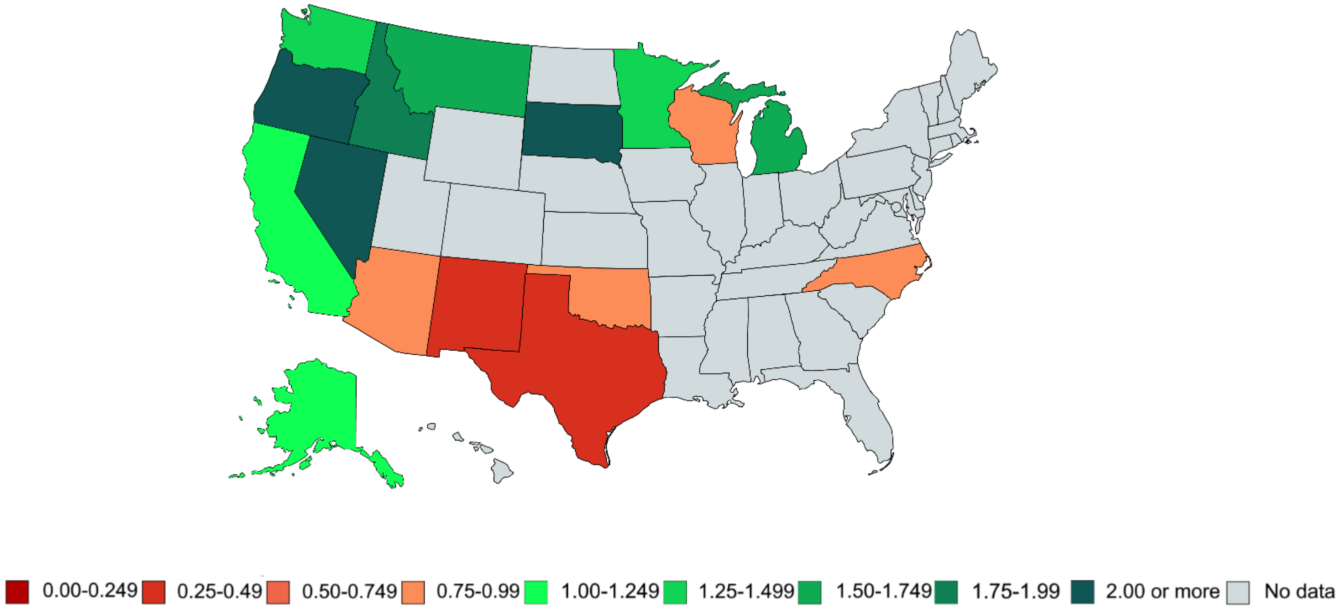

SOURCE: Authors' analysis of Medicare claims data from a 20% random sample of Part D enrollees with fee-for-service coverage, restricted to older adult beneficiaries aged 65+ years with at least 1 outpatient claim with an OUD diagnosis or opioid overdose in any study year (2015-2019).

NOTES: Appendix exhibit A15 shows color-coded map of the United States with each state shaded in based on the average percent of older adult American Indian/Alaska Native beneficiaries who received buprenorphine conditional on an OUD or overdose diagnosis between 2015-2019 relative to that of their White counterparts. Darker shades of red indicate smaller ratios and greater disparities, while states shaded in green indicate more equal ratios of buprenorphine receipt in a given state. States where the racial/ethnic group has <50 total individuals across the study period are shaded gray and indicated as 'no data' on maps.

Appendix Exhibit A16. State-level ratio of percentage of Asian/Pacific Islander patients with OUD receiving buprenorphine to White patients with OUD receiving buprenorphine, 65+, 2015-2019

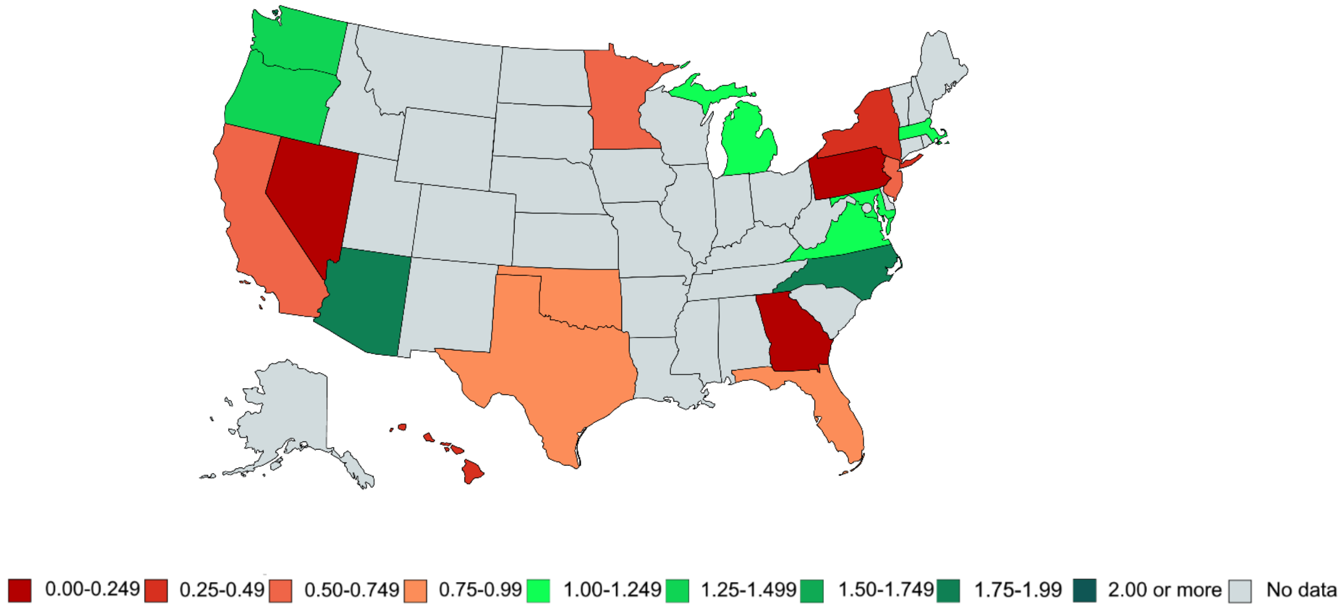

SOURCE: Authors' analysis of Medicare claims data from a 20% random sample of Part D enrollees with fee-for-service coverage, restricted to older adult beneficiaries aged 65+ years with at least 1 outpatient claim with an OUD diagnosis or opioid overdose in any study year (2015-2019).

NOTES: Appendix exhibit A16 shows color-coded map of the United States with each state shaded in based on the average percent of older adult Asian/Pacific Islander beneficiaries who received buprenorphine conditional on an OUD or overdose diagnosis between 2015-2019 relative to that of their White counterparts. Darker shades of red indicate smaller ratios and greater disparities, while states shaded in green indicate more equal ratios of buprenorphine receipt in a given state. States where the racial/ethnic group has <50 total individuals across the study period are shaded gray and indicated as 'no data' on maps.

Appendix Exhibit A17. Logistic regression of buprenorphine receipt on year and race/ethnicity among Medicare older adult beneficiaries with OUD or opioid overdose, 2015-2019 (N = 343,955)

**Variable**	**aOR**	**95% CI**	**p-value**
Year	1.29	1.27-1.30	<.001
Race/ethnicity			
White	REF	REF	REF
American Indian/Alaska Native	0.69	0.42-1.13	.140
Asian/Pacific Islander	0.65	0.39-1.07	.088
Black	0.88	0.78-0.99	.035
Hispanic	0.69	0.56-0.84	<.001
Age	0.92	0.91-0.92	<.001
Male sex	1.61	1.55-1.67	<.001
Enrolled in Medicaid	1.00	0.96-1.05	.885
Interactions			
Year*White	REF	REF	REF
Year*American Indian/Alaska Native	1.19	1.04-1.36	.012
Year*Asian/Pacific Islander	1.08	0.95-1.24	.239
Year* Black	0.96	0.92-0.99	.010
Year*Hispanic	0.99	0.93-1.05	.713

SOURCE: Authors' analysis of Medicare claims data from a 20% random sample of Part D enrollees with fee-for-service coverage, restricted to older adult beneficiaries aged 65+ years with at least 1 claim with an OUD or opioid overdose diagnosis in any study calendar year (2015-2019).

NOTES: Estimates are from an individual-level logistic regression model with standard errors clustered on beneficiary ID and fixed effect for state. aOR = adjusted odds ratio; CI = confidence interval

Appendix Exhibit A18. Multilevel logistic regression of buprenorphine receipt on year and race/ethnicity among Medicare older adult beneficiaries with OUD or opioid overdose, sensitivity analysis using CCW OUD definition, 2015-2019 (N = 90,151)

**Variable**	**aOR**	**95% CI**	**p-value**
Year	1.34	1.31-1.37	<.001
Race/ethnicity			
White	REF	REF	REF
American Indian/Alaska Native	0.92	0.46-1.85	.815
Asian/Pacific Islander	0.80	0.35-1.86	.608
Black	1.11	0.95-1.31	.190
Hispanic	1.05	0.81-1.35	.716
Age	0.92	0.92-0.93	<.001
Male sex	1.56	1.44-1.67	<.001
Enrolled in Medicaid	0.91	0.84-0.99	.022
Interactions			
Year*White	REF	REF	REF
Year*American Indian/Alaska Native	1.22	0.99-1.50	.065
Year*Asian/Pacific Islander	1.13	0.89-1.43	.319
Year* Black	0.93	0.88-0.97	.002
Year*Hispanic	0.95	0.88-1.03	.193

SOURCE: Authors' analysis of Medicare claims data from a 20% random sample of Part D enrollees with fee-for-service coverage, restricted to older adult beneficiaries aged 65+ years with an OUD diagnosis or opioid overdose based on the Chronic Condition Data Warehouse definition of opioid use disorder (1 inpatient *or* 2 outpatient claims with an OUD or opioid overdose diagnosis) in any study year (2015-2019).

NOTES: Estimates are from an individual-level logistic regression model with standard errors clustered on beneficiary ID and fixed effect for state. aOR = adjusted odds ratio; CI = confidence interval

Appendix Exhibit A19. Multilevel logistic regression of buprenorphine receipt on year and race/ethnicity among Medicare older adult beneficiaries with OUD or opioid overdose, sensitivity analysis excluding beneficiaries dually enrolled in Medicaid, 2015-2019 (N = 331,295)

**Variable**	**aOR**	**95% CI**	**p-value**
Year	1.25	1.23-1.27	<.001
Race/ethnicity			
White	REF	REF	REF
American Indian/Alaska Native	0.89	0.40-1.95	.765
Asian/Pacific Islander	0.58	0.29-1.15	.117
Black	0.98	0.80-1.20	.839
Hispanic	0.64	0.45-0.92	.016
Age	0.92	0.91-0.92	<.001
Male sex	1.59	1.51-1.68	<.001
Interactions			
Year*White	REF	REF	REF
Year*American Indian/Alaska Native	1.14	0.97-1.42	.239
Year*Asian/Pacific Islander	1.17	0.87-0.98	.109
Year* Black	0.93	0.92-1.14	.013
Year*Hispanic	1.03	1.23-1.27	.626

SOURCE: Authors' analysis of Medicare claims data from a 20% random sample of Part D enrollees with fee-for-service coverage, restricted to non-dual eligible older adult beneficiaries aged 65+ years with at least 1 claim with an OUD or opioid overdose diagnosis in any study calendar year (2015-2019).

NOTES: Estimates are from an individual-level logistic regression model with standard errors clustered on beneficiary ID and fixed effect for state. aOR = adjusted odds ratio; CI = confidence interval

Appendix Exhibit A20. Adjusted ratios of percentage of older adult beneficiaries with OUD or opioid overdose who received buprenorphine relative to White older adult beneficiaries, 2015-2019

**Variable**	**Adjusted Ratio**	**95% CI**
2015		
White	REF	REF
American Indian/Alaska Native	0.69	0.37-1.29
Asian/Pacific Islander	0.62	0.32-1.20
Black	1.14	0.99-1.31
Hispanic	0.82	0.65-1.04
2016		
White	REF	REF
American Indian/Alaska Native	0.87	0.58-1.33
Asian/Pacific Islander	0.71	0.45-1.10
Black	1.09	0.98-1.21
Hispanic	0.80	0.67-0.95
2017		
White	REF	REF
American Indian/Alaska Native	0.99	0.70-1.39
Asian/Pacific Islander	0.77	0.54-1.09
Black	1.05	0.95-1.14
Hispanic	0.79	0.68-0.92
2018		
White	REF	REF
American Indian/Alaska Native	1.18	0.92-1.51
Asian/Pacific Islander	0.81	0.62-1.07
Black	1.00	0.92-1.08
Hispanic	0.78	0.69-0.89
2019		
White	REF	REF
American Indian/Alaska Native	1.38	1.14-1.68
Asian/Pacific Islander	0.88	0.70-1.10
Black	0.96	0.90-1.03
Hispanic	0.77	0.69-0.86

SOURCE: Authors’ analysis of Medicare claims data from a 20% random sample of Part D enrollees with fee-for-service coverage, restricted to older adult beneficiaries aged 65+ years with at least 1 claim with an OUD or opioid overdose diagnosis in any study calendar year (2015-2019).

NOTES: Adjusted ratios and CIs use predicted probabilities within each year and racial/ethnic group from an individual-level logistic regression model shown in Appendix Exhibit A11. aOR = adjusted odds ratio; CI = confidence interval

Appendix Exhibit A21: Unadjusted state percentage of older adult beneficiaries with OUD or opioid overdose who received buprenorphine, 2015-2019

State	White %	Black %	Hispanic %	Asian/Pacific Islander %	American Indian/Alaska Native %
National – All States	4.6	4.5	3.3	1.8	3.6
Alabama	5.3	2.1	NR	NR	NR
Alaska	7.8	NR	NR	NR	9.1
Arizona	3.0	2.0	2.7	5.6	2.8
Arkansas	1.7	0.6	NR	NR	NR
California	6.8	3.9	2.9	3.7	7.6
Colorado	4.3	1.9	3.0	NR	NR
Connecticut	7.4	6.0	7.7	NR	NR
Delaware	2.3	2.8	2.1	NR	NR
District of Columbia	10.1	19.1	NR	NR	NR
Florida	4.2	1.4	1.1	3.6	NR
Georgia	3.8	3.3	0.9	0.0	NR
Hawaii	9.7	NR	0.0	3.4	NR
Idaho	3.5	NR	5.4	NR	7.0
Illinois	4.2	6.6	4.5	NR	NR
Indiana	5.2	4.0	6.9	NR	NR
Iowa	1.2	0.0	1.9	NR	NR
Kansas	1.9	0.0	2.2	NR	NR
Kentucky	3.7	0.4	NR	NR	NR
Louisiana	3.1	1.3	3.8	NR	NR
Maine	8.6	NR	NR	NR	NR
Maryland	4.5	10.1	4.6	5.3	NR
Massachusetts	11.3	14.7	21.4	11.6	NR
Michigan	6.1	6.0	5.9	6.8	10.7
Minnesota	3.0	3.5	1.6	1.8	4.1
Mississippi	2.5	1.7	5.8	NR	NR
Missouri	3.1	2.3	0.0	NR	NR
Montana	2.4	NR	NR	NR	4.1
Nebraska	3.7	2.7	NR	NR	NR
Nevada	3.5	0.9	2.0	0.8	18.6
New Hampshire	5.4	NR	NR	NR	NR
New Jersey	5.6	4.6	3.9	2.9	NR
New Mexico	4.2	9.2	4.0	NR	1.3
New York	6.1	5.0	4.5	1.6	NR
North Carolina	4.2	3.9	4.5	7.4	3.8
North Dakota	0.8	NR	NR	NR	NR
Ohio	5.0	7.3	8.4	NR	NR
Oklahoma	3.7	2.7	2.4	3.3	3.4
Oregon	7.4	6.4	6.3	9.6	16.0
Pennsylvania	3.7	4.1	7.6	0.0	NR
Rhode Island	13.7	NR	NR	NR	NR
South Carolina	4.4	2.1	NR	NR	NR
South Dakota	4.1	NR	NR	NR	10.5
Tennessee	2.2	1.5	2.4	NR	NR
Texas	2.9	2.6	1.6	2.2	1.0
Utah	6.4	NR	9.3	NR	NR

Vermont	8.7	NR	NR	NR	NR
Virginia	3.8	2.4	3.7	4.1	NR
Washington	5.6	5.8	5.5	7.3	8.0
West Virginia	5.1	6.8	NR	NR	NR
Wisconsin	3.4	4.2	11.4	NR	3.0
Wyoming	6.7	NR	NR	NR	NR

SOURCE: Authors’ analysis of Medicare claims data from a 20% random sample of Part D enrollees with fee-for-service coverage, restricted to older adult beneficiaries aged 65+ years with at least 1 claim with an OUD or opioid overdose diagnosis in any study calendar year (2015-2019).

NOTES: Racial/ethnic groups with <50 total individuals in the state across the study period are not reported (NR) due to small cell sizes.

Appendix Exhibit A22: Unadjusted state ratios of percentage of beneficiaries with OUD or opioid overdose who received buprenorphine relative to White older adult beneficiaries, 2015-2019

**State**	**Black**	**Hispanic**	**Asian/Pacific Islander**	**American Indian/Alaska Native**
	**Ratio (95% CI)**	**Ratio (95% CI)**	**Ratio (95% CI)**	**Ratio (95% CI)**
National – All States	1.00 (0.96-1.04)	0.78 (0.73-0.83)	0.81 (0.70-0.94)	1.13 (1.00-1.29)
Alabama	0.39 (0.27-0.56)	NR	NR	NR
Alaska	NR	NR	NR	1.17 (0.69-1.98)
Arizona	0.67 (0.28-1.6)	0.91 (0.59-1.41)	1.87 (0.72-4.88)	0.91 (0.41-2.03)
Arkansas	0.36 (0.11-1.13)	NR	NR	NR
California	0.57 (0.49-0.67)	0.42 (0.37-0.49)	0.54 (0.43-0.69)	1.12 (0.79-1.57)
Colorado	0.44 (0.16-1.16)	0.71 (0.47-1.07)	NR	NR
Connecticut	0.81 (0.55-1.2)	1.04 (0.71-1.53)	NR	NR
Delaware	1.24 (0.77-1.99)	0.94 (0.23-3.78)	NR	NR
District of Columbia	1.88 (1.15-3.09)	NR	NR	NR
Florida	0.33 (0.23-0.47)	0.26 (0.17-0.38)	0.87 (0.4-1.91)	NR
Georgia	0.89 (0.69-1.16)	0.24 (0.03-1.71)	0.00	NR
Hawaii	NR	0.00	0.35 (0.16-0.81)	NR
Idaho	NR	1.54 (0.69-3.42)	NR	2 (0.76-5.22)
Illinois	1.58 (1.33-1.89)	1.09 (0.72-1.65)	NR	NR
Indiana	0.78 (0.57-1.07)	1.34 (0.65-2.75)	NR	NR
Iowa	0.00	1.58 (0.22-11.25)	NR	NR
Kansas	0.00	1.2 (0.3-4.82)	NR	NR
Kentucky	0.12 (0.03-0.48)	NR	NR	NR
Louisiana	0.41 (0.29-0.59)	1.24 (0.59-2.59)	NR	NR
Maine	NR	NR	NR	NR
Maryland	2.27 (1.98-2.6)	1.02 (0.49-2.13)	1.19 (0.54-2.61)	NR
Massachusetts	1.3 (1.1-1.55)	1.9 (1.62-2.23)	1.03 (0.54-1.98)	NR
Michigan	0.98 (0.87-1.11)	0.96 (0.62-1.47)	1.11 (0.54-2.27)	1.74 (0.9-3.36)
Minnesota	1.19 (0.68-2.08)	0.55 (0.08-3.9)	0.62 (0.09-4.32)	1.4 (0.63-3.12)
Mississippi	0.68 (0.5-0.92)	2.32 (0.89-6.06)	NR	NR
Missouri	0.73 (0.43-1.25)	0.00	NR	NR
Montana	NR	NR	NR	1.72 (0.85-3.47)
Nebraska	0.74 (0.18-2.94)	NR	NR	NR
Nevada	0.25 (0.11-0.56)	0.58 (0.32-1.06)	0.22 (0.03-1.55)	5.38 (3.1-9.34)
New Hampshire	NR	NR	NR	NR
New Jersey	0.83 (0.71-0.96)	0.7 (0.55-0.88)	0.53 (0.29-0.98)	NR
New Mexico	2.17 (0.99-4.77)	0.95 (0.67-1.33)	NR	0.3 (0.04-2.11)
New York	0.82 (0.69-0.98)	0.73 (0.58-0.92)	0.27 (0.09-0.82)	NR
North Carolina	0.93 (0.77-1.14)	1.08 (0.55-2.14)	1.78 (0.82-3.85)	0.92 (0.5-1.69)
North Dakota	NR	NR	NR	NR
Ohio	1.46 (1.19-1.78)	1.69 (1.04-2.77)	NR	NR
Oklahoma	0.73 (0.49-1.07)	0.63 (0.3-1.33)	0.88 (0.22-3.45)	0.9 (0.67-1.22)
Oregon	0.86 (0.42-1.77)	0.85 (0.53-1.38)	1.30 (0.56-3.00)	2.16 (1.37-3.4)
Pennsylvania	1.12 (0.88-1.42)	2.06 (1.45-2.92)	0.00	NR
Rhode Island	NR	NR	NR	NR
South Carolina	0.47 (0.29-0.78)	NR	NR	NR
South Dakota	NR	NR	NR	2.59 (1.11-6.03)
Tennessee	0.68 (0.42-1.11)	1.09 (0.28-4.29)	NR	NR
Texas	0.87 (0.71-1.07)	0.53 (0.42-0.68)	0.75 (0.36-1.57)	0.34 (0.05-2.37)
Utah	NR	1.46 (0.86-2.48)	NR	NR
Vermont	NR	NR	NR	NR

Virginia	0.63 (0.44-0.9)	0.98 (0.37-2.58)	1.08 (0.36 -3.29)	NR
Washington	1.04 (0.73-1.48)	0.97 (0.68-1.41)	1.30 (0.82-2.07)	1.42 (0.99-2.05)
West Virginia	1.33 (0.72-2.47)	NR	NR	NR
Wisconsin	1.25 (0.79-1.96)	3.36 (2.13-5.31)	NR	0.88 (0.22-3.48)
Wyoming	NR	NR	NR	NR

SOURCE: Authors' analysis of Medicare claims data from a 20% random sample of Part D enrollees with fee-for-service coverage, restricted to disability beneficiaries aged 18-64 years with at least 1 claim with an OUD or opioid overdose diagnosis in any study calendar year (2015-2019).

NOTES: Racial/ethnic groups with <50 total individuals in the state across the study period are not reported (NR) due to small cell sizes. Ratios are calculated as the percentage of individuals in each minoritized racial/ethnic group who received buprenorphine divided by the percentage of white individuals who received buprenorphine across the study period (2015-2019). CI= Confidence Interval
